# Dysregulated PI3K Signaling in B Cells of CVID Patients

**DOI:** 10.3390/cells11030464

**Published:** 2022-01-28

**Authors:** Ina Harder, Matthias Münchhalfen, Geoffroy Andrieux, Melanie Boerries, Bodo Grimbacher, Hermann Eibel, Maria Elena Maccari, Stephan Ehl, Jürgen Wienands, Julia Jellusova, Klaus Warnatz, Baerbel Keller

**Affiliations:** 1 Department of Rheumatology and Clinical Immunology, Medical Center—University of Freiburg, Faculty of Medicine, University of Freiburg, Freiburg, Germany; ina.harder@uniklinik-freiburg.de (I.H.); hermann.eibel@uniklinik-freiburg.de (H.E.); 2 Center for Chronic Immunodeficiency (CCI), Medical Center—University of Freiburg, Faculty of Medicine, University of Freiburg, Freiburg, Germany; 3 Institute of Cellular and Molecular Immunology, University Medical Center Göttingen, Göttingen, Germany; matthias.muenchhalfen@med.uni-goettingen.de (M.M.); jwienan@gwdg.de (J.W.); 4 Institute of Medical Bioinformatics and Systems Medicine, Medical Center—University of Freiburg, Faculty of Medicine, University of Freiburg, Freiburg, Germany; geoffroy.andrieux@uniklinik-freiburg.de (G.A.); melanie.boerries@uniklinik-freiburg.de (M.B.); 5 German Cancer Consortium (DKTK), Partner site Freiburg, German Cancer Research Center (DKFZ), Heidelberg, Germany; 6 Institute for Immunodeficiency, Center for Chronic Immunodeficiency (CCI), Medical Center, Faculty of Medicine, Albert-Ludwigs-University of Freiburg, Freiburg, Germany; bodo.grimbacher@uniklinik-freiburg.de (B.G.); maria.elena.maccari@uniklinik-freiburg.de (M.E.M.); stephan.ehl@uniklinik-freiburg.de (S.E.); 7 Clinic of Rheumatology and Clinical Immunology, Center for Chronic Immunodeficiency (CCI), Medical Center, Faculty of Medicine, Albert-Ludwigs-University of Freiburg, Freiburg, Germany; 8 DZIF—German Center for Infection Research, Satellite Center Freiburg, Freiburg, Germany; 9 CIBSS—Centre for Integrative Biological Signalling Studies, Albert-Ludwigs University, Freiburg, Germany; 10 RESIST—Cluster of Excellence 2155 to Hanover Medical School, Satellite Center Freiburg, Freiburg, Germany; 11 Division of Pediatric Hematology and Oncology, Department of Pediatrics and Adolescent Medicine, Medical Center–University of Freiburg, Faculty of Medicine, University of Freiburg, Freiburg, Germany; 12 Institute of Clinical Chemistry and Pathobiochemistry, School of Medicine, Klinikum Rechts der Isar, Technical University of Munich, 81675 Munich, Germany; julia.jellusova@tum.de; 13 TranslaTUM, Center for Translational Cancer Research, Technical University of Munich, 81675 Munich, Germany

**Keywords:** CVID, B cells, immune dysregulation, PI3K, mTOR, S6, FOXO1, BCR signaling, CD21^low^ B cells, APDS

## Abstract

The altered wiring of signaling pathways downstream of antigen receptors of T and B cells contributes to the dysregulation of the adaptive immune system, potentially causing immunodeficiency and autoimmunity. In humans, the investigation of such complex systems benefits from nature’s experiments in patients with genetically defined primary immunodeficiencies. Disturbed B-cell receptor (BCR) signaling in a subgroup of common variable immunodeficiency (CVID) patients with immune dysregulation and expanded T-bet^high^CD21^low^ B cells in peripheral blood has been previously reported. Here, we investigate PI3K signaling and its targets as crucial regulators of survival, proliferation and metabolism by intracellular flow cytometry, imaging flow cytometry and RNAseq. We observed increased basal but disturbed BCR-induced PI3K signaling, especially in T-bet^high^CD21^low^ B cells from CVID patients, translating into impaired activation of crucial downstream molecules and affecting proliferation, survival and the metabolic profile. In contrast to CVID, increased basal activity of PI3K in patients with a gain-of-function mutation in *PIK3CD* and activated PI3K delta syndrome (APDS) did not result in impaired BCR-induced AKT-mTOR-S6 phosphorylation, highlighting that signaling defects in B cells in CVID and APDS patients are fundamentally different and that assessing responses to BCR stimulation is an appropriate confirmative diagnostic test for APDS. The active PI3K signaling in vivo may render autoreactive T-bet^high^CD21^low^ B cells in CVID at the same time to be more sensitive to mTOR or PI3K inhibition.

## 1. Introduction

Common variable immunodeficiency (CVID) comprises a heterologous group of patients with primary antibody deficiency. Only for some patients have genetic defects been described to date. CVID is defined by low serum IgG and IgA with or without IgM and a poor vaccination response or differentiation of class-switched memory B cells [1]. Besides recurrent bacterial infections, especially of the respiratory and the gastrointestinal tract, nearly half of CVID patients present with a variety of non-infectious manifestations of immune dysregulation. These comprise autoimmune cytopenias, granulomatous disease, interstitial lung disease, splenomegaly, lymphadenopathy and chronic enteropathy [2,3,4]. CVID patients with immune dysregulation often display a more severe reduction of switched memory B cells and an accumulation of T-bet^high^CD21^low^ B cells in peripheral blood [5,6,7]. The underlying mechanism of B-cell dysfunction and immune dysregulation varies and frequently remains elusive. 

T-bet^high^CD21^low^ B cells typically accumulate in the context of chronic immune activation in CVID, autoimmune diseases such as systemic lupus erythematosus (SLE) and rheumatoid arthritis (RA), chronic infections by HIV, HCV or *Plasmodium falciparum* and others [8,9,10,11,12,13]. The expansion of this B-cell population depends on antigen-induced B-cell receptor (BCR)- as well as T-cell-derived IFNγ-, CD40- and IL-21-signals resulting in a unique transcriptional profile including high levels of the transcription factor T-bet [14,15,16,17,18,19]. We and others have reported disturbed BCR signaling at the level of SYK, BLNK, PLCγ2, reduced Ca^2+^ mobilization and NF-κB activation in CD21^low^ B cells and homologous populations in different disease conditions [10,20,21,22,23,24,25,26,27,28]. Furthermore, the CD21^pos^ B cells of CVID patients also showed alterations in SYK phosphorylation, canonical NF-κB signaling as well as Ca^2+^ mobilization, which were less pronounced compared to the CD21^low^ B-cell compartment [23,24,27].

The phosphoinositide-3-kinase (PI3K) and its downstream targets AKT, mTOR and FOXO1 are central players in B-cell biology [29,30]. This signaling pathway is crucial in fundamental processes such as in proliferation, survival, metabolism, class switch recombination and differentiation to plasma cells [31,32]. Four different isoforms of class I PI3Ks are expressed in B cells: PI3Kα, -β, -δ and -γ, which are constituted of the catalytic subunits p110α, p110β and p110δ, coupled to the regulatory subunit p85 and p110γ bound to regulatory p101 or p84 [30]. Murine models have shown that, while p110β seems to play a minor role in B-cell biology, p110γ is predominantly activated by G protein-coupled receptors, especially mediating chemokine-mediated downstream signaling [29,30]. P110α and p110δ show some redundancy during pre B-cell development, and both isoforms contribute to tonic BCR signaling; however, BCR-activated PI3K signaling primarily depends on p110δ, while p110α is less involved [29,30,33]. The necessity of tightly balanced regulation of PI3K activity is also illustrated in patients with activated PI3Kδ syndrome (APDS): overactive PI3Kδ signaling leads to a variable degree of humoral immunodeficiency, impaired control of EBV infection, lymphoproliferation and lymphoma and some immune dysregulation [34,35,36,37]. Gain of function mutations (GOF) in *PIK3CD* encoding p110δ (APDS1) or loss of function (LOF) mutations in *PIK3R1* encoding p85α (APDS2) are associated with increased phosphorylation of AKT and subsequent targets as the ribosomal subunit S6 in lymphocytes [34,38]. Increased proportions of transitional B cells and T-bet^high^CD21^low^ B cells and a loss of class-switched memory B cells have been described [17,36,37,39,40]. Thus, some APDS patients present clinically and immunologically in a similar way as CVID.

Given the crucial role of PI3K in B-cell signaling and physiology and the described phenotypical overlap, we set out to analyze this pathway in patients with CVID of unknown genetic origin. We observed increased basal but disturbed BCR-activated PI3K signaling especially but not only in the CD21^low^ B cells of these patients. Although increased basal phosphorylation resembles APDS-derived B cells, impaired activation-induced PI3K signaling in CVID clearly distinguishes both disease entities. 

## 2. Materials and Methods

Patient cohort and ethics statement: Blood from patients and controls was obtained under local ethics approval protocols (Freiburg 251/13). Informed consent was obtained from all patients, patients’ parents or healthy donors according to the declaration of Helsinki. Overall, 45 patients diagnosed with CVID were analyzed, including 18 male and 27 female individuals with a median age of 44 years (range 19–78 years). Four patients presented with an infection-only CVID phenotype and 41 patients presented with immune dysregulation. Five patients diagnosed with APDS were included, three male and two female individuals with a median age of 13 years (range 6–36 years).

Cell isolation: PBMCs were isolated from EDTA blood by Ficoll density centrifugation following standard protocols. Given the concordance of CD21^low^CD38^low^ B cells and T-bet^high^CD21^low^ B cells [17] in the following, T-bet^high^CD21^low^ B cells were defined by CD21^low^CD38^neg^ and referred to as CD21^low^ B cells. Naïve B cells were isolated using the naïve B cell isolation kit (Miltenyi Biotech, Bergisch Gladbach, Germany) following the manufacturer’s instructions. For some experiments, sorted naïve CD27^neg^CD38^low/intermediate^ and naïve CD27^neg^CD38^neg^ CD21^low^ B cells were used.

Cell cultivation and activation: Survival of B-cell subpopulations was determined after cultivation of 10.000 sorted CD21^low^ and CD21^pos^ B cells in RPMI 10% FCS for 48 h. Proliferative marker Ki67 was determined after stimulation of 0.5 × 10^6^ PBMCs with CpG (cat. # tlrl-2006-1, Invivogen, San Diego, CA, USA) and anti-IgM (cat. #202201, SouthernBiotech, Birmingham, AL, USA) or IL-4 (cat. #11340043, Immunotools, Friesoythe, Germany), anti-CD40 (cat. #MAB6321, R&D Systems, Minneapolic, MN, USA) and anti-IgM for 48 h. Induction of HIF1α and GLUT1 was determined after stimulation of PBMCs with anti-IgM (cat. #202214, Southern Biotech, Birmingham, AL, USA) for 40 h. 

Antibodies and dyes used in this study: CD3 PE-Cy7 (UCHT1 cat. #300420), CD4 BV421 (RPA-T4 cat. #300532), CD10 BV605 (HI10a cat. #312222), CD19 BV421 (HIB19) (cat. #302234), CD19 APC-Cy7 (HIB19 cat. #302218), CD21 PE-Cy7 (Bu32 cat. #354912), CD27 BV421 (M-T271 cat. #356418), CD38 PerCp-Cy5.5 (HIT2 cat. #303522), CD38 APC (HIT2 cat. #303510), IgD BV785 (IA6-2 cat. #348242), IgM APC-Cy7 (MHM-88 cat. #314520), IgM BV421 (MHM-88 cat. #314516), Lambda FITC (MHL-38 cat. #316606), HIF1α PE (546-16 cat. #359703), KI67 AF488 (ki67 cat. #350507), Zombie UV (cat. #423107) (all BioLegend, San Diego, CA); IgD FITC (cat. #203202), IgD PE (cat. #203209), IgA PE (cat. #205009) (all Southern Biotech, Birmingham, AL); IgM AF647 (cat. #109606129), IgM AF488 (cat. #109545129 (both Jackson ImmunoResearch, West Grove, PA, USA), CD21 PE (BL13) (cat. #A32536), (both Beckman Coulter, Brea, CA); Glut1 AF647 (EPR3915) (cat. #ab195020), Mitotracker Red CMXRos (cat. #ab176832) (both Abcam, Cambridge, UK); CD27 BV605 (L128 cat. #562655), S6(pS235/pS236) AF647 (N7-548 cat. #560435), IgG AF700 (G18-145 cat. #G18145), Annexin V PE (cat. #5165875X) (all BD Biosciences, La Jolla, CA, USA); mTOR(pS2448) PE (MRRBY cat. #12971842) (ebioscience, San Diego, CA, USA); AKT (pS473) AF488 (D9E cat. #4071S), FOXO1 PE (C29H4 cat. #14262), goat anti-rabbit AF647 (cat. #4414) (Cell Signaling Technologies, Frankfurt, Germany); GLUT1 rabbit (cat. #NB300666) (Novus Biologicals, Centennial, CO, USA); 2-NBDG (cat. #N13195) (Invitrogen, Carlsbad, CA, USA); CD19(pY531) rabbit (cat. #ab63443) (Abcam, Cambridge, UK).

Flow cytometry and cell sorting: Standard surface staining was performed at 4° C in the dark for 15 min. Ex vivo staining of GLUT1 was performed on 1 × 10^6^ PBMCs at 37 °C for 1 h, followed by secondary goat anti-rabbit AF647 staining for 20 min and surface staining. Glucose uptake was determined after incubation of 1 × 10^6^ PBMCs with fluorescent 2-NBDG at 37 °C for 1 h, mitochondrial mass was evaluated by incubation of 1 × 10^6^ PBMCs with Mitotracker Red CMX at 37 °C for 20 min followed by cell surface staining. Intracellular staining of signaling molecules was performed as follows: for signaling assays, 0.5 × 10^6^ freshly isolated PBMCs were rested at 37° C in IMDM 10% FCS for 60 min. Frozen APDS samples (APDS1 #1 and APDS2 #2) were thawed and rested for 3 h at 37° before stimulation. If not mentioned differently, phosphorylation of AKT, mTOR and S6 was determined after stimulation with 15 µg/mL F(ab’)^2^ anti-IgM (Southern Biotech, Birmingham, AL, USA) for 45 min, CD40L at optimal concentrations for 30 min or 5 µg/mL CpG (Invivogen, San Diego, CA, USA) for 90 min. pCD19 was analyzed after stimulation with anti-IgM for 3 min. After fixation at 37 °C for 10 min with Cytofix and permeabilization with ice cold Perm III (both BD Biosciences, La Jolla, CA, USA) on ice for 30 min, cells were stained with the respective antibodies for 30 min at room temperature in the dark. Ki67 was determined using the Intraprep kit (Beckman Coulter, Brea, CA, USA) following the manufacturers’ instructions. Survival was addressed by Annexin V and DAPI staining of cultivated, sorted CD21^pos^ and CD21^low^ populations following the manufacturers’ instructions. The counts of living AnnexinV^neg^DAPI^neg^ cells were determined by flow cytometry. Data were acquired on a LSR Fortessa or a Canto II (both BD Biosciences, La Jolla, CA, USA). Cells were sorted on a MoFlo Astrios Cell sorter (Beckman Coulter, Brea, CA, USA).

Flow cytometry and imaging: Nuclear localization of FOXO1 in subsets of primary human naïve B cells was addressed using multicolor imaging flow cytometry. 0.5–3 × 10^6^ isolated naïve B cells (purity 60–94%) were rested for 60 min at 37 °C and subsequently stimulated with anti-κ for 60 min at 37 °C. Fixation occurred by addition of Cytofix and incubation for 20 min at 4 °C. Samples were centrifuged at 700× *g* for 8 min at RT and washed twice with ice-cold PBS 0.3% TritonX100 and PBS 2% FCS 0.3% TritonX100, respectively. Upon washing, cells were resuspended in 100 μL PBS 2% FCS 0.3% TritonX100 containing all antibodies for surface and intracellular staining. After incubation for 45 min at 4 °C, cells were washed twice with PBS 2% FCS and stored overnight at 4 °C. After washing with PBS, nuclei were stained by resuspension in 50 µL 10 µg/mL 7-AAD (7-aminoactinomycin D, Thermo Fisher, Waltham, MA, USA) in PBS. 

Per sample, at least 5000 single, focused cells were recorded (as determined via area vs. aspect ratio and gradient RMS, respectively) at the Amnis ImageStreamX MkII imaging flow cytometer (Luminex, Austin, TX, USA). Images were acquired in 60× magnification, and the following channels were used: Ch1: brightfield, Ch2: Lambda FITC, Ch3: FOXO1 PE, Ch5: 7-AAD, Ch6: CD21 PE-Cy7, Ch7 IgM BV421, Ch9: brightfield, Ch11: CD38 APC, Ch12: SSC. Fluorophore compensation and statistical analysis were performed using the IDEAS software. Cells were gated as depicted in the respective figure. Nuclear localization was assessed using the “Nuclear Translocation Wizard” software feature on the channels 3 (anti-FOXO1-PE) and 5 (7-AAD). Events with a “SimilarityDilate” (co-localization score) >1.0 were considered cells with nuclear-localized FOXO1.

RNAseq: RNA of 50,000–275,000 sorted naïve CD21^pos^ B cells from CVID patients and HD and patient-derived CD21^low^ B cells were isolated and processed for RNAseq as described before [17]. An amount of 10 ng of RNA (RIN as determined by Bioanalyzer; Agilent Technologies, Franfurt, Germany: 7.9–9.9) was loaded. Differential analysis was performed using the limma R package [41]. The generally applicable gene set enrichment (GAGE) R package was used to identify the upregulated and downregulated gene sets based on MSigDB annotation [42]. For both analyses, adjusted *p* value (Benjamini and Hochberg) below 0.05 was considered as significant. 

Statistics: Statistical analysis was performed using Graphpad Prism 9 (GraphPad Software Inc., San Diego, CA, USA). Normal distribution was determined by D’Agostino and Pearson test, or for low sample numbers by Shapiro–Wilk test. Data were analyzed using paired *t*-test, one-way ANOVA with Tukey’s multiple comparisons test or Kruskal–Wallis with Wilcoxon post hoc tests.

*p* values below 0.05 were considered significant. * *p* < 0.05, ** *p* < 0.01, *** *p* < 0.001, **** *p* < 0.0001.

## 3. Results

### 3.1. AKT, mTOR and S6 Phosphorylation in B-Cell Subpopulations of CVID Patients 

**Figure 1:**
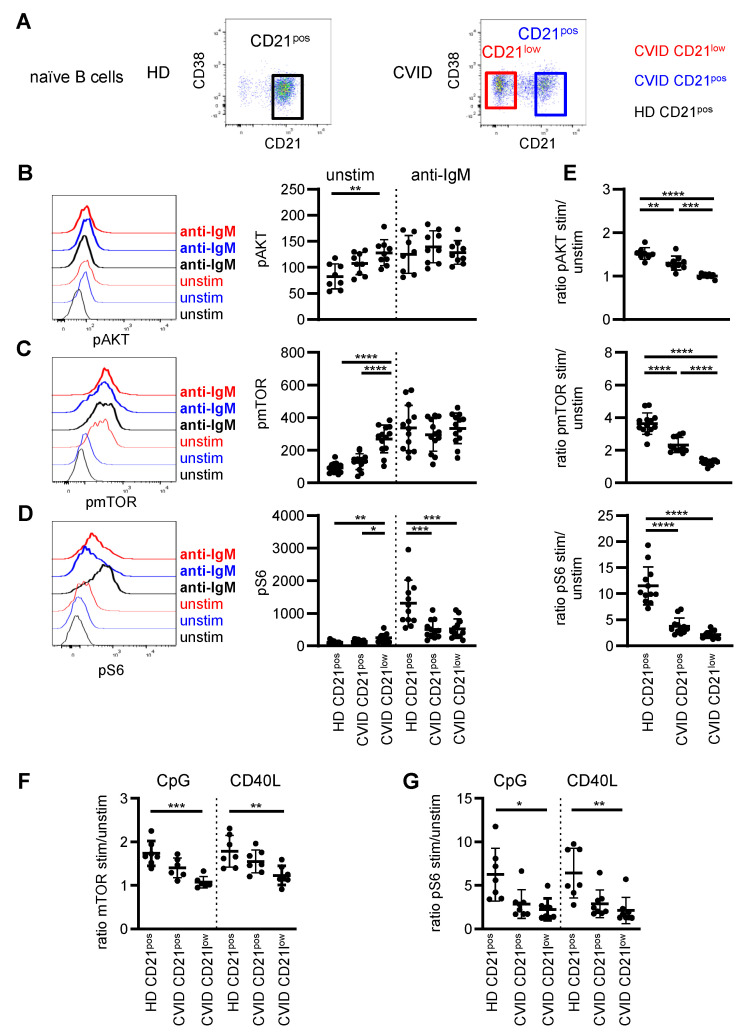
Disturbed phosphorylation of AKT, mTOR and S6 in naïve B cells of CVID patients. (**A**) CD21^pos^ B cells in one representative HD and CD21^pos^ and CD21^low^ B cells in one representative CVID patient as determined by CD21 and CD38 expression after gating on IgM^pos^CD27^neg^ CD19^pos^ B cells. (**B**) Representative histogram of pAKT in populations as described and statistics of the MFI of pAKT unstimulated and after stimulation with anti-IgM for 45 min in CVID patients (*n* = 9) and HD (*n* = 8). (**C**) Representative histogram and statistics as described in (**A**) for pmTOR (*n* = 13) and (**D**) pS6 (*n* = 12). (**E**) Ratio of the MFI of pAKT, pmTOR and pS6 after stimulation to unstimulated as shown in (**B**–**D**). (**F**) Ratio of the MFI of pmTOR and (**G**) pS6 stimulated with CpG or CD40L to unstimulated in CD21^pos^ B cells of HD and CD21^pos^ and CD21^low^ B cells from CVID patients (*n* = 7–8). * *p* < 0.05, ** *p* < 0.01, *** *p* < 0.001, **** *p* < 0.0001.

To visualize the PI3K signaling pathway, phosphorylation of AKT and the downstream effector molecules mTOR and ribosomal protein S6 were analyzed in B cells of CVID patients and healthy donors (HD) in the unstimulated situation and after BCR triggering. To ensure optimal comparability of patient and HD-derived B cells, analysis was restricted to the IgM^pos^CD27^neg^ naïve B-cell compartment, and in the following CD21^low^ and CD21^pos^ B cells refer to the respective naïve B-cell subsets (Figure 1A and see Supplementary Figure S1A for the full gating strategy). Given the low proportions of CD21^low^ B cells in most healthy individuals, this population was not further investigated in HD [17]. 

Basal phosphorylation of AKT, mTOR and S6 (Figure 1B–D) was significantly increased in patients’ CD21^low^ B cells compared to CD21^pos^ B cells of HD. Slightly increased though not significant levels of pAKT and pmTOR were also observed for patients’ CD21^pos^ B-cell compartment. Upon BCR stimulation, the phosphorylation of AKT, mTOR and S6 significantly increased in all analyzed B-cell subsets except for AKT in CD21^low^ B cells, which could not be further increased (Supplementary Figure S1B). After anti-IgM stimulation, the mean fluorescence intensity (MFI) of phosphorylated AKT and mTOR was overall comparable in B-cell subsets of CVID patients and HD (Figure 1B,C); however, phosphorylation of S6 was significantly reduced in patients’ CD21^low^ and CD21^pos^ B cells (Figure 1D). Noticeably, the fold increase of phosphorylated AKT and mTOR after stimulation to unstimulated samples was intermediately reduced in CD21^pos^ B cells and almost absent in the CD21^low^ B cells of CVID patients (Figure 1E). In contrast, BCR-induced phosphorylation of S6 was severely compromised in CVID patients’ CD21^pos^ and CD21^low^ B-cell subsets (Figure 1E). This disturbance in S6 phosphorylation was not caused by delayed signaling, as prolonged BCR stimulation for 16 h did not restore phosphorylation of S6 in both patient-derived B-cell subsets (Supplementary Figure S1C). 

To evaluate if altered PI3K signaling was BCR-specific, CD21^pos^ B cells of HD, CD21^pos^ and CD21^low^ B cells of CVID patients were analyzed after incubation with CD40 ligand (CD40L) and CpG activating toll-like receptor 9 (TLR9). Activation-induced phosphorylation of mTOR after both stimuli was significantly reduced in CD21^low^ B cells of CVID patients, with intermediate though not significant changes in the patients’ CD21^pos^ B cells (Figure 1F). In accordance with BCR stimulation, activation-induced phosphorylation of S6 was low in both patient-derived B-cell subsets after CpG and CD40L stimulation (Figure 1G). 

Overall, these data indicate that the PI3K signaling pathway was generally altered in the CVID patients’ B cells, with more pronounced alterations in CD21^low^ B cells.

### 3.2. Expression of Molecules Involved in PI3K Signaling

**Figure 2:**
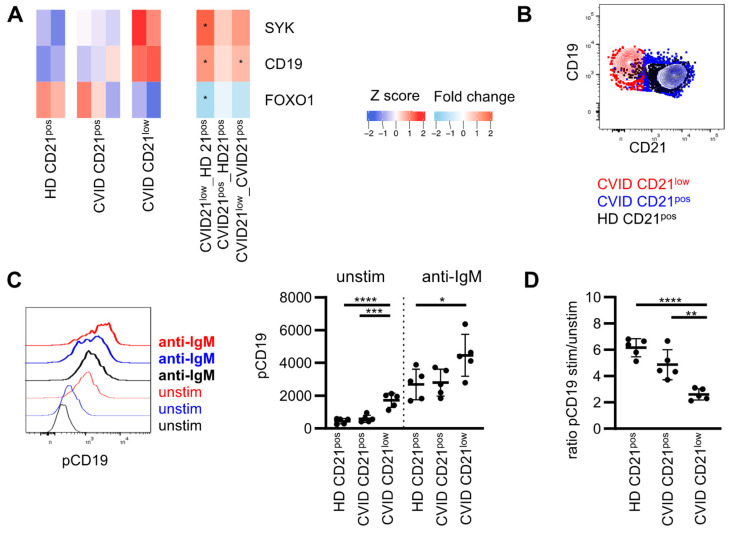
Adaptor protein CD19 is pre-phosphorylated in naïve CD21^low^ B cells. (**A**) Heat map showing selected differentially expressed genes (DEGs) in CD21^low^ B cells compared to CD21^pos^ in HD involved in PI3K signaling downstream of the BCR. Stars indicate significant dysregulation. Fold changes (FC) between the different subpopulations as indicated. (**B**) Representative overlay showing CD21 and CD19 in CD21^low^ and CD21^pos^ B cells of a CVID patient. (**C**) Representative histogram of pCD19 in populations as described and statistics of the MFI of pCD19 unstimulated and after stimulation with anti-IgM in CVID patients (*n* = 5) and HD (*n* = 5). (**D**) Ratio of the MFI of pCD19 stimulated and unstimulated as described in (**C**). * *p* < 0.05, ** *p* < 0.01, *** *p* < 0.001, **** *p* < 0.0001.

RNAseq data of the respective B-cell subsets at a resting state were analyzed regarding the expression of genes encoding for proteins, which are involved in PI3K signaling. Transcripts of all PI3K catalytic subunits were present in the analyzed B-cell populations with the highest expression of *PIK3CD* and the lowest expression of *PIK3CB* (Supplementary Figure S2A). Expression of *PIK3CB* and *PIK3CG* was significantly reduced in CD21^low^ B cells compared to CD21^pos^ B cells from HD, overall implying a minor role of both isoforms for the described alterations in basal and induced AKT, mTOR and S6 phosphorylation. *PIK3CA* was similarly expressed in CD21^pos^ and CD21^low^ B cells, but three log scales were reduced compared to *PIK3CD* (Supplementary Figure S2A). A regulatory subunit of class I PI3Ks, *PIK3R1*, was equally expressed, as seen for *PIK3R5*, whereas *PIK3R6* was increased in CD21^low^ B cells (Supplementary Figure S2A). As previously reported, *SYK* and *CD19* were increased in CD21^low^ B cells compared to HD (Figure 2A) [8,23,43]. In turn, the expression of the transcription factor *FOXO1* was reduced, while the expression of other genes involved in this pathway was not significantly altered (Supplementary Figure S2B). CD19 is part of the BCR co-receptor complex recruiting PI3K. Increased CD19 protein expression was associated with increased basal CD19 phosphorylation in CD21^low^ B cells, which is a prerequisite for PI3K recruitment [44] (Figure 2B,C). Phosphorylation was substantially increased in CD21^low^ B cells upon BCR stimulation, reaching overall higher levels compared to CD21^pos^ B cells (Figure 2C). The fold increase stimulated and unstimulated was significantly reduced in CD21^low^ B cells compared to CD21^pos^ B cells in patients and HD (Figure 2D). As noted before, an intermediate though not significant reduction was observed in CVID patients’ CD21^pos^ B-cell compartment (Figure 2D). 

### 3.3. Downstream Signaling und Target Genes

**Figure 3:**
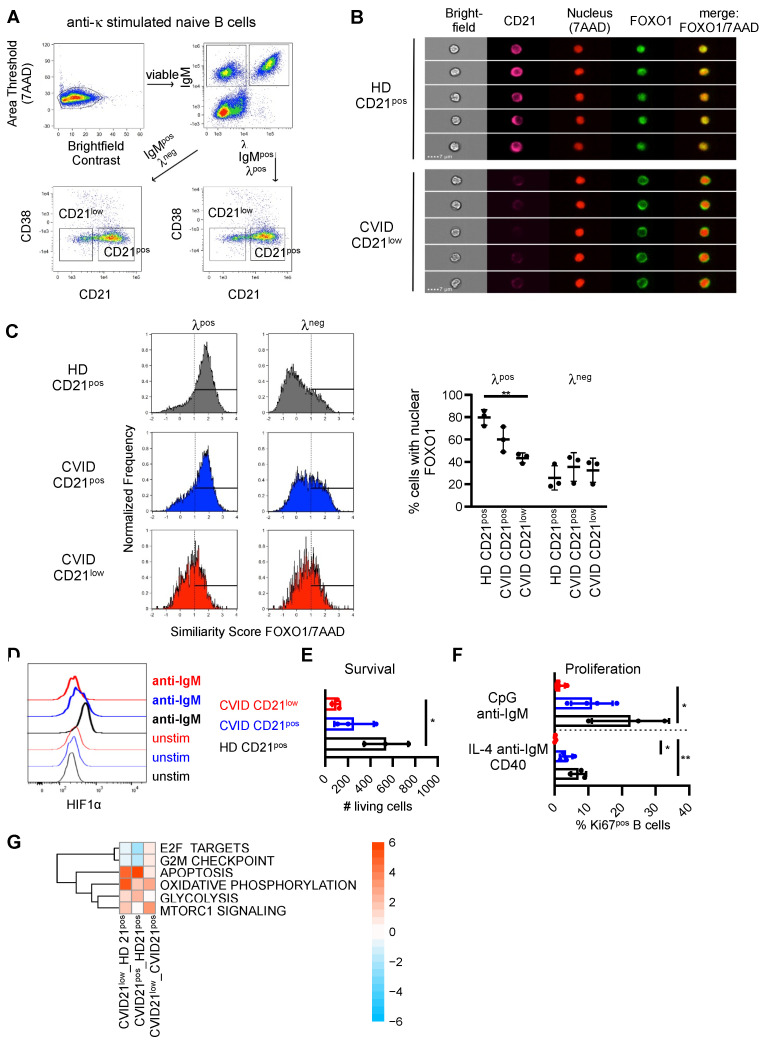
Disturbed signaling downstream of PI3K in CVID patients’ B cells. (**A**) Gating strategy for imaging and multicolor flow cytometry for nuclear FOXO1. Anti-κ-stimulated naïve B cells were stained for λ, IgM, CD21 and CD38 to identify IgM^pos^, λ^pos^ and λ^neg^ CD21^pos^ and CD21^low^ B cells. Gating on λ^pos^ B cells determined unstimulated populations, λ^neg^ cells represented κ-stimulated populations. (**B**) Representative images of λ^pos^CD21^pos^ B cells of HD and λ^pos^CD21^low^ B cells of a CVID patient. (**C**) Representative histograms of the similarity score of FOXO1 and 7AAD in λ^pos^ and λ^neg^ CD21^pos^ B cells of HD, CD21^pos^ and CD21^low^ B cells in CVID patients and statistics of the percentage of cells with nuclear FOXO1. (**D**) Expression of HIF1α in populations as indicated unstimulated and after stimulation with anti-IgM for 40 h. Data are representative for one of four independent experiments. (**E**) Numbers of living AnnexinV^neg^DAPI^neg^ CD21^low^ and CD21^pos^ B cells after incubation of sorted cells for 48 h (*n* = 3–4). (**F**) Proportion of KI67^pos^, CD21^low^ and CD21^pos^ B cells after stimulation (*n* = 3). (**G**) Significantly altered hallmark pathways in CD21^low^ or CD21^pos^ B cells of CVID compared to CD21^pos^ B cells of HD as determined by RNAseq of B-cell subsets ex vivo. * *p* < 0.05, ** *p* < 0.01.

Activation of AKT by PI3K leads to the phosphorylation of the transcription factor FOXO1 and the initiation of a negative feedback loop by which phosphorylated FOXO1 is translocated from the nucleus to the cytoplasm. The localization of FOXO1 was analyzed in CD21^low^ and CD21^pos^ B cells by flow cytometry and imaging in isolated naïve B cells after stimulation of the BCR with anti-κ (Figure 3A). λ^pos^ B cells were evaluated as unstimulated controls. In line with our findings of increased basal phosphorylation of AKT and reduced BCR-mediated phosphorylation, nuclear levels of FOXO were reduced in unstimulated λ^pos^ CD21^low^ B cells, and BCR-mediated shuttling into the cytosol was almost absent in patients’ λ^neg^ κ-stimulated CD21^low^ B cells (Figure 3B,C). Furthermore, in the unstimulated situation, nuclear FOXO1 tended to be lower in CVID-derived CD21^pos^ B cells compared to the CD21^pos^ counterparts in HD (Figure 3C). 

Protein expression of the PI3K-pathway target gene HIF1α was upregulated in naïve CD21^pos^ B cells of HD after BCR stimulation for 40 h (Figure 3D). Compatible with impaired PI3K signaling upon activation, BCR-mediated upregulation of HIF1α expression was reduced in CD21^pos^ B cells and almost abrogated in CD21^low^ B cells of CVID patients (Figure 3D). 

PI3K signaling is critically linked to the survival and proliferation of B cells [45,46]. The numbers of living cells were significantly reduced in sorted CD21^low^ B cells from CVID patients compared to the CD21^pos^ B cells of healthy individuals (Figure 3E). CD21^pos^ B cells of CVID patients displayed intermediate numbers of living cells, which did not reach significance. Upon stimulation, induction of the proliferation marker Ki67 tended to be lower in the CD21^pos^ B-cell compartment of CVID patients and was significantly reduced in patients’ CD21^low^ B cells, indicating impaired proliferation of this B-cell subset (Figure 3F).

In line with our functional data, RNAseq data of B-cell subpopulations analyzed ex vivo revealed significantly enriched hallmark pathways mTORC1 signaling and apoptosis in the B cells of CVID patients, whereas cell cycle progression and DNA replication were reduced, as indicated by decreased expression of G2M checkpoint and E2F targets (Figure 3G). Consistent with the regulatory role of AKT and mTOR in the metabolic programming of immune cells [46], genes involved in oxidative phosphorylation and glycolysis were enriched in patients’ B-cell subpopulations (Figure 3G). 

Thus, despite increased basal activation, the induction of downstream pathways of PI3K signaling was compromised in CVID patients’ naïve B-cell subsets, with the most pronounced alterations in the CD21^low^ B cells of patients. 

### 3.4. Metabolic Characteristics

**Figure 4:**
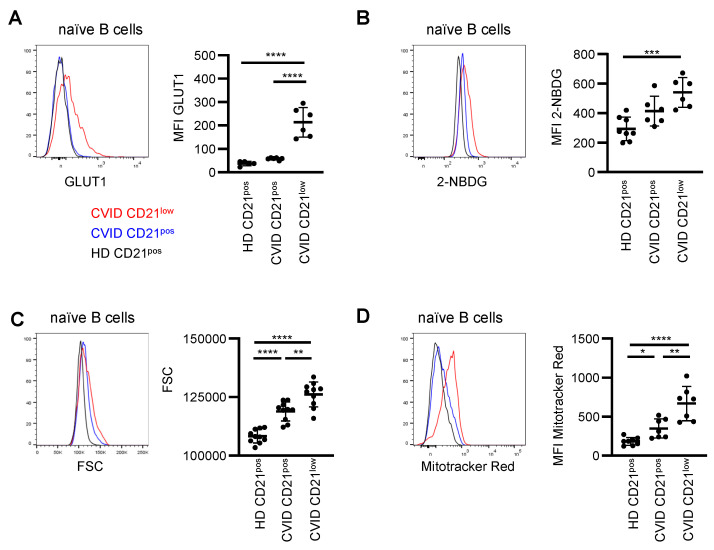
Enhanced metabolic activity in B cells of CVID patients. (**A**) Representative histogram of GLUT1 expression in CD21^pos^ B cells from HD (black) and in CD21^pos^ (blue) and CD21^low^ (red) B cells from CVID patients. Statistical analysis of the relative expression of GLUT1 compared to the respective day control (*n* = 11). (**B**) Representative histogram for 2-NBDG and statistics thereof (*n* = 6–8). (**C**) Representative histogram overlay and statistics of the FSC (*n* = 10). (**D**) Representative histogram overlay displaying MitoTracker Red and statistics of the MFI (*n* = 7–8). * *p* < 0.05, ** *p* < 0.01, *** *p* < 0.001, **** *p* < 0.0001.

Basal activation of PI3K pathway signaling was increased in CVID patients’ CD21^low^ B cells, with minor changes in the CD21^pos^ B-cell compartment, and oxidative phosphorylation and glycolysis-related signatures were enriched. As PI3K/AKT/mTOR activity is a critical regulator of metabolic activity [46], and glucose is a crucial supplier of energy, we analyzed the cellular capacity for glucose uptake. We observed significantly increased basal expression of the glucose transporter GLUT1 in the CD21^low^ B cells of CVID patients (Figure 4A). In line with the previous notion of impaired activation of PI3K-induced target genes, BCR-induced upregulation of GLUT1 was severely reduced in naïve B cells of CVID patients (Supplementary Figure S3). In line, the uptake of the fluorescent glucose analog 2-NBDG was increased, especially in the CD21^low^ B cells of CVID patients compared to the CD21^pos^ B cells of HD (Figure 4B), indicating enhanced glucose acquisition. Furthermore, CD21^pos^ and even more pronounced CD21^low^ B cells of CVID patients exhibited significantly increased cell size (Figure 4C) and increased mitochondrial mass, as determined by MitoTracker Red (Figure 4D), compared to the CD21^pos^ B cells from HD. An increase in cell size and mitochondrial mass is consistent with elevated basal mTOR activity in the CD21^low^ B-cell compartment. Overall, these data indicate enhanced metabolic capacity, especially in CVID patients’ CD21^low^ B cells. 

### 3.5. Correlation with Clinical and Immunological Features

While PI3K signaling was commonly disturbed in CD21^low^ B cells, CD21^pos^ B cells showed intermediate changes in AKT and mTOR phosphorylation, more pronounced disturbance in S6 phosphorylation and overall intermediate changes in downstream pathways and functions. However, the degree varied between individual patients. To address these differences, the signaling capacity of CVID patients’ CD21^pos^ B cells upon BCR stimulation was correlated with the percentage of CD21^low^ B cells in the respective patient (Supplementary Figure S4A). We did not observe a correlation of CD21^low^ B cell proportion with the ratio of mTOR and S6 phosphorylation. 

Furthermore, patients were divided regarding the occurrence of non-infectious manifestations of immune dysregulation. No differences in signaling capacity of AKT, mTOR and S6 were observed between patients with autoimmune manifestations, autoimmune cytopenia, lymphoproliferation, granuloma formation or splenomegaly (Supplementary Figure S4B), indicating that none of the complications can be directly associated with a particular signaling profile within the group of CVID patients. 

### 3.6. Delineation from Activated PI3Kδ Syndrome (APDS)

GOF mutations in PI3K signaling in APDS patients gave us the opportunity to compare the direct effects of a genetically defined overactive PI3Kδ with the observed signaling alterations in CVID. This is of special interest, as especially AKT and S6 phosphorylation are used in diagnostics to confirm the relevance of variants of unknown significance (VUS) in *PIK3CD*. Five patients harboring previously described activating mutations in *PIK3CD* encoding p110δ or *PIK3R1* encoding p85α were analyzed.

**Figure 5:**
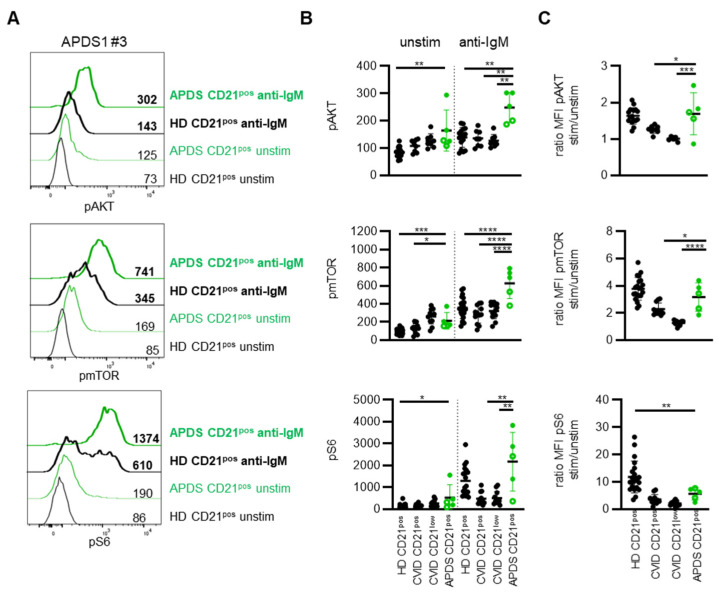
PI3K signaling differentiates between APDS and CVID patients. (**A**) Histogram overlays of pAKT, pmTOR and pS6 in CD21^pos^ B cells of one APDS patient (green) and the respective HD (black) unstimulated (normal lines) and after stimulation with anti-IgM (bold lines). (**B**) Statistics of the MFI of pAKT, pmTOR and pS6 unstimulated and after anti-IgM stimulation in CD21^pos^ B cells of HD, CD21^pos^ and CD21^low^ B cells of CVID patients (all depicted in black) and in CD21^pos^ B cells of APDS patients (green). Patients with GOF mutations in p110δ (filled circles) and LOF mutations in p85α (open circles) were differentiated. (**C**) Ratio of the MFI of pAKT, pmTOR and pS6 after anti-IgM stimulation and unstimulated as shown in (**B**). * *p* < 0.05, ** *p* < 0.01, *** *p* < 0.001, **** *p* < 0.0001.

Although the level of pre-activation tended to be more pronounced in patients with *PIK3CD* GOF mutations compared to *PIK3R1* LOF mutations, basal phosphorylation of AKT, mTOR and S6 in CD21^pos^ B cells was elevated for all analyzed APDS1 and APDS2 patients compared to the respective day control (Figure 5A and Supplementary Figure S5A). Statistical analysis revealed significantly increased phosphorylation for all three molecules compared to an extended cohort of HD; however, basal phosphorylation of AKT and S6 was not significantly different compared to CVID patient-derived CD21^pos^ and remarkably similar to CD21^low^ B cells for most of the APDS patients (Figure 5B). Phosphorylation of mTOR was significantly higher in the CD21^pos^ B cells of APDS compared to the CD21^pos^ B cells of CVID patients but did not clearly separate between both groups (Figure 5B). In contrast to the limited differences of CVID- and APDS-derived B cells concerning basal activation, BCR-mediated phosphorylation of AKT and mTOR in APDS the patients’ CD21^pos^ B cells was significantly increased compared to HD as well as CVID patients’ CD21^pos^ and CD21^low^ B cells (Figure 5B and Supplementary Figure S5). Interestingly, phosphorylation of S6 was not significantly elevated compared to HD after BCR stimulation (Figure 5B and Supplementary Figure S5). Although strikingly increased in some patients, the MFI of phosphorylated S6 was even reduced in one APDS2 patient compared to the respective day control, indicating that phosphorylation of the ribosomal protein may be more sensitive for extrinsic factors. For AKT and mTOR, this translated into significantly increased ratios compared to the CVID patients’ B cells but not compared to HD (Figure 5C). 

Thus, although increased pre-activation is common in B cells of CVID and APDS patients, activation-induced phosphorylation was markedly increased for AKT and mTOR and may be used for the discrimination of commonly observed changes in CVID and genetically determined signaling differences in APDS.

## 4. Discussion

Activity of the PI3K pathway provides fundamental regulatory signals in B-cell biology. Tonic PI3K signaling delivers survival signals predominantly via PI3Kα and PI3Kδ, and induced PI3K signaling primarily transduced by PI3Kδ conveys proliferation and activation [29,30,45]. CD21^low^ B cells presented with signs of enhanced basal PI3K activity and downstream targets, whereas BCR-, CD40- or TLR-mediated activation of this pathway was compromised. These observations parallel previous notions of pre-phosphorylation but reduced BCR-induced activation of SYK, BTK and PLCγ2 in these cells and homologous populations in different disease conditions [21,22,23,26,27]. We and others have also shown that increased expression of SYK in these cells contributes to the pre-activation of SYK, BTK and PLCγ2 [23,43]. Since SYK phosphorylation is upstream of PI3K activation in B cells [47], elevated SYK protein expression may also feed into the increased basal activation of PI3K signaling ex vivo. The transcriptome analysis allows one to speculate that the altered expression of other components of the PI3K signaling pathway may also contribute to the observed altered activity of constitutive and induced PI3K signaling. This will need further exploration in the future when the adequate tools for analysis in primary human B cells become available. This is similarly true for the investigation of RAS proteins, another pathway relevant for the activation of PI3K proteins [30]. Furthermore, CD21^low^ B cells displayed not only high levels of CD19 protein expression but also phosphorylation. Phosphorylated CD19 is the central adaptor for PI3K recruitment [48], well compatible with an association of high CD19 expression and enhanced basal phosphorylation of PI3K signaling in CD21^low^ B cells. 

At the same time, the profound increase in basal activation may actually contribute to impaired PI3K signaling upon BCR stimulation in these cells, as aberrant basal signaling associated with reduced activation-induced signaling has been observed in B-cell lymphoma and malignancies [49]. On the other hand, high SYK expression did not generally impair BCR signaling [23] and overexpression of CD19-augmented BCR signaling in mice [48]. Most importantly, here we could show that in contrast to CVID patients’ B cells, increased PI3K activity, especially but not exclusively in APDS1, led not only to increased basal phosphorylation but translated into even enhanced phosphorylation of AKT and mTOR after BCR stimulation. These observations demonstrate that the pure increase in baseline activity of certain signaling molecules does not necessarily lead to impaired signaling after BCR activation. Preserved BCR-induced PI3K pathway activation actually delineates signaling in CVID and APDS and implies additional factors causing the poor BCR-induced PI3K signal in CVID patients’ B cells. A complex network of dysregulated proteins, including kinases but also inhibitory receptors and associated phosphatases [27,50,51], most likely contributes to the signaling alterations observed in CD21^low^ and to a milder degree in the CD21^pos^ B cells of CVID patients. 

Patient-derived CD21^pos^ B cells displayed intermediate changes for PI3K signaling, which we also reported for other signal proteins downstream of the BCR [23,24,27]. Given the intermediate gene expression pattern and an increased proportion of T-bet^pos^ cells among naïve CD21^pos^ B cells of CVID patients [17], it is tempting to speculate that this population comprises cells, which are prone to develop, or started differentiation towards a CD21^low^ phenotype, including the characteristic signaling pattern. Interestingly, despite minor changes in the activation of upstream molecules such as AKT and mTOR in the CD21^pos^ B cells of CVID patients, BCR-induced phosphorylation of the ribosomal protein S6 was severely reduced in both CD21^pos^ and CD21^low^ B cells from CVID patients. Phosphorylation of S6 by S6K is activated by mTOR complex 1 (mTORC1) but also by PI3K-independent mechanisms such as amino acid availability, energy or oxygen supply [52,53]. Determining the role of these factors in the severe disturbance of S6 phosphorylation in both naïve B-cell subsets derived from CVID patients requires further investigations. 

Altered PI3K signaling in patients’ B cells was associated with increased apoptosis, reduced proliferation after in vitro stimulation and changes in the metabolic state ex vivo. Interestingly, although all of these functions are regulated by the PI3K pathway [46], the intermediate signaling alterations seen in patients’ CD21^pos^ B cells did not always correlate with the functional changes, pointing towards a more complex interaction and integration of multiple different signaling pathways including NF-κB, Ca^2+^, MAPK and others, which derive from different receptors [54,55]. However, B-cell metabolism seems to be more tightly linked to PI3K signaling [56]. RNAseq data of CD21^low^ B cells ex vivo indicated enhanced expression of genes involved in oxidative phosphorylation and glycolysis compared to HD, which fits to the observed pre-activation in this cell type and corroborates data from HIV-derived CD21^low^ homologues [57]. In line with recent reports showing that enhanced mTORC1 signaling leads to increased cell mass and mTOR signaling drives mitochondrial biogenesis [58], we noted increased cell size and mitochondrial mass especially in CD21^low^ B cells and to a lower extent in the CD21^pos^ B cells of CVID patients. It is, however, noteworthy that enhanced metabolic capacity ex vivo did not result in increased proliferation or differentiation in vitro. Additional metabolic changes after BCR activation are needed for in vitro proliferation and differentiation [31,59], which may be compromised in patients’ B cells. Enhanced basal activity of PI3K signaling in CVID patients’ CD21^low^ B cells was associated with decreased levels of nuclear FOXO1 and increased basal expression of the transcription factor HIF1α, most likely reflecting the constitutively activated phenotype of this B-cell population [20,21,26,60,61]. This lack of active FOXO1 potentially contributes to impaired class switching [62], whereas disturbed regulation of FOXO1 after activation in these cells may result in impaired cycle progression and proliferation, commonly associated with an export of this transcription factor from the nucleus [63]. In line with constitutive activation of CD21^low^ B cells, B-cell activation can induce hypoxia-related HIF-1α in an oxygen-independent manner [64]. However, HIF-1α-mediated *IL10* gene expression (data not shown) or a metabolic shift to glycolysis [64] compared to the CD21^pos^ B cells of CVID patients was not observed in CD21^low^ B cells. We hypothesize that the broad dysregulation of these transcription factors and others in the unstimulated situation and disturbed dynamic regulation of these factors upon activation contribute to the functional impairment of CD21^low^ B cells. 

Corroborating our observations, alterations in the PI3K pathway such as enhanced basal pAKT, increased cytoplasmic FOXO and reduced PTEN activity, an antagonist of PI3K signaling, have been reported in the B cells of SLE patients, frequently presenting with an accumulation of CD21^low^ B cells [61,65,66]. Wu et al. described hyper-activated mTORC1 and increased basal phosphorylation of mTOR and S6 to be crucial for the generation of a CD21^low^ B-cell homologue in SLE [67]. Thus, PI3K or downstream mTOR inhibition may represent a therapeutic option to delete or to prevent the development of this cell type with increased auto-reactive BCR specificities [12,20]. 

Here, we demonstrated that basal as well as induced PI3K signaling, which regulates the metabolic state, survival, activation and differentiation of this lymphocyte lineage, is severely altered in the naïve B cells of CVID patients. In addition to previous reports [68,69], we found increased baseline but impaired BCR-mediated PI3K pathway activation, especially in the CD21^low^ B cells of CVID patients, providing potential therapeutic options to target these cells by specific inhibitors of PI3Kδ or mTOR. Altered PI3K signaling may well also contribute to the immunodeficiency in CVID as PI3K signal strength, and the dynamic regulation of transcription factors, such as FOXO1 and HIF1α, determines the selection into short-lived plasmablasts or germinal center B-cell fate, the induction of somatic hyper mutation and class switch recombination and finally the integrity of memory and plasma cell formation [54,55,62,70,71]. It remains to be understood whether alterations in the required rewiring of the PI3K signaling network downstream of CD40 and the BCR and dynamic activity of mTORC1 is disturbing selection and thereby contributes to the observed autoimmunity in these patients [70,72]. Our study also demonstrated that the presence of GOF mutations in *PIK3CD* does not impair BCR-induced AKT, mTOR and S6 phosphorylation, rendering this assay a very helpful confirmatory diagnostic test for APDS in the differential diagnosis from other CVID patients.

## Data Availability

RNAseq data of CD21^low^ and CD21^pos^ B cells of CVID patients and HD are available at NCBI GEO under accession GSE148163.

## Supplementary Materials

The following are available online at https://www.mdpi.com/article/10.3390/cells11030464/s1, Figure S1: Gating strategy and PI3K signaling in B-cell subpopulations; Figure S2: PI3K signaling components; Figure S3: Upregulation of GLUT11 in B-cell subpopulations; Figure S4: Correlation of PI3K signaling with proportions of CD21low B cells and manifestations of immune dysregulation in CVID; Figure S5: pAKT, pmTOR and pS6 in APDS patients. Supplementary Figure Legends.
