# Supplementary material for: Dysregulated PI3K Signaling in B Cells of CVID Patients
Source: Cells. 2022 Jan 28;11(3):464. doi: 10.3390/cells11030464 (PMC8834633; doi:10.3390/cells11030464)
Supplement: Supplementary file 1 [file cells-11-00464-s001.zip › Supplementary Figure Legends PI3K.pdf]

cells MDPI

Supplementary Figure Legends

Supplementary Figure S1. Gating strategy and PI3K signaling in B-cell subpopulations (A) Gating Strategy for $CD21^{pos}$ in one representative HD and $CD21^{low}$ and $CD21^{pos}$ in one representative CVID patient after gating on $CD19^{pos}CD21^{pos/low}$ and $CD27^{neg}IgM^{pos}$ naïve B cells. **(B)** Statistics of the MFI of pAkt, pmTOR and pS6 unstimulated and after anti-IgM stimulation in $CD21^{pos}$ B cells from HD, $CD21^{pos}$ and $CD21^{low}$ B cells from CVID patients (pAKT, HD n=8, CVID n=9; pmTOR, HD n=13, CVID n=13; pS6, HD n=13, CVID n=13). **(C)** Statistical analysis of the MFI of pS6 unstimulated and after anti-IgM stimulation for 16 hours in $CD21^{pos}$ B cells from HD (n=4) and $CD21^{pos}$ and $CD21^{low}$ B cells from CVID patients (n=4) and ratio of the MFI stimulated and unstimulated.

Supplementary Figure S2. Expression of PI3K signaling components Heat map of selected genes involved in PI3K signaling in B cells as determined by RNAseq in $CD21^{pos}$ B cells from HD (n=2), $CD21^{pos}$(n=3) and $CD21^{low}$ B cells (n=2) from CVID patients. Fold changes (FC) between the different subpopulations as indicated. Stars indicate significant differences.

Supplementary Figure S3. Upregulation of GLUT1 in B-cell subpopulations (A) Representative histogram overlays for GLUT1 in $CD21^{pos}$ B cells from HD, $CD21^{pos}$ and $CD21^{low}$ B cells from CVID patients without stimulation or after stimulation with anti-IgM for 40 hours. (B) Ratio of the MFI of GLUT1 after anti-IgM stimulation and unstimulated as described in (A) in $CD21^{pos}$ B cells from HD (n=3), $CD21^{pos}$ (n=3) and $CD21^{low}$ (n=3) B cells from CVID patients.

Supplementary Figure S4. Correlation of PI3K signaling with proportions of $CD21^{low}$ B cells and manifestations of immune dysregulation in CVID (A) Correlation of the proportions of $CD21^{low}$ B cells of B cells and ratios of pmTOR (n=15) and pS6 (n=26) in $CD21^{pos}$ B cells from CVID patients. **(B)** Statistical analysis of the ratio of pAKT, pmTOR and pS6 stimulated and unstimulated after grouping of patients according to non-infectious manifestations (AI=autoimmunity, AC= autoimmune cytopenia, LP=lymphoproliferation, G=granuloma or Spl=splenomegaly).

Supplementary Figure S5. pAKT, pmTOR and pS6 in APDS patients (A) Histogram overlays of pAKT, pmTOR and pS6 in $CD21^{pos}$ B cells from three APDS1 patients and two APDS2 patients (green) with the corresponding daily controls (black) unstimulated (normal lines) and after anti-IgM Stimulation (bold lines). Numbers indicate the MFI of the signaling protein under the respective condition.
