# Supplementary material for: Dysregulated PI3K Signaling in B Cells of CVID Patients
Source: Cells. 2022 Jan 28;11(3):464. doi: 10.3390/cells11030464 (PMC8834633; doi:10.3390/cells11030464)

Supplementary Figure 1

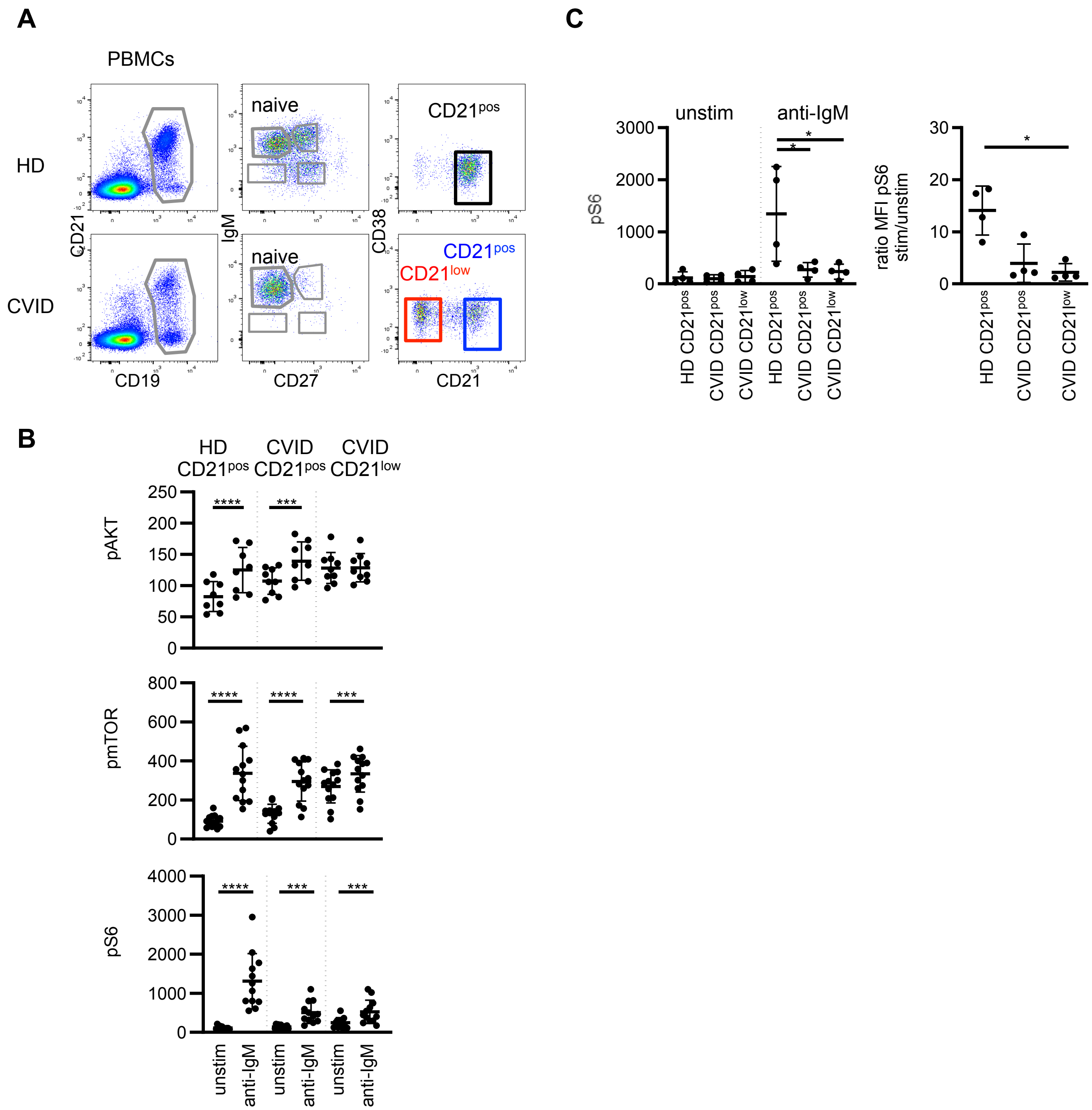

Supplementary Figure 2

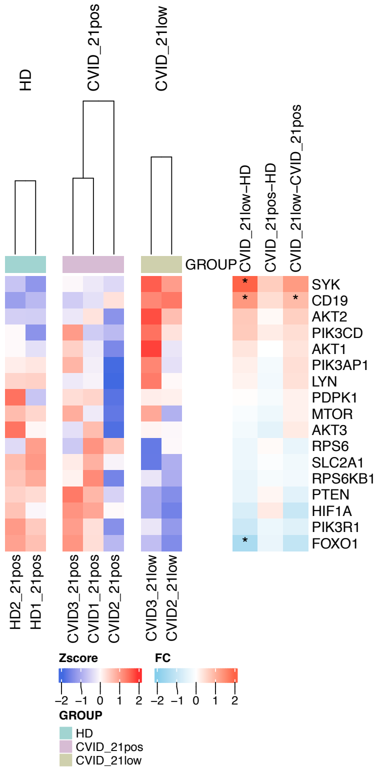

Supplementary Figure 3

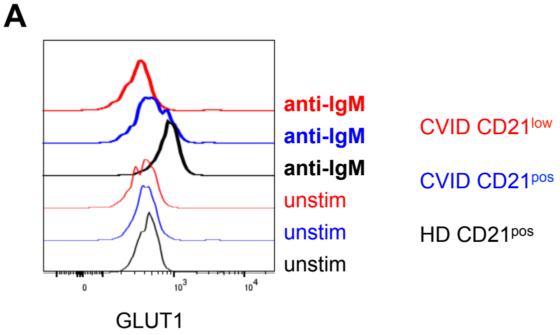

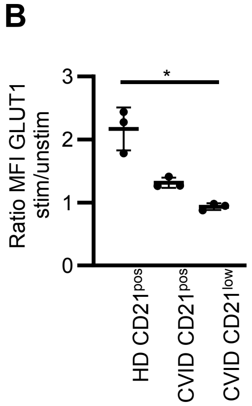

Supplementary Figure 4

A

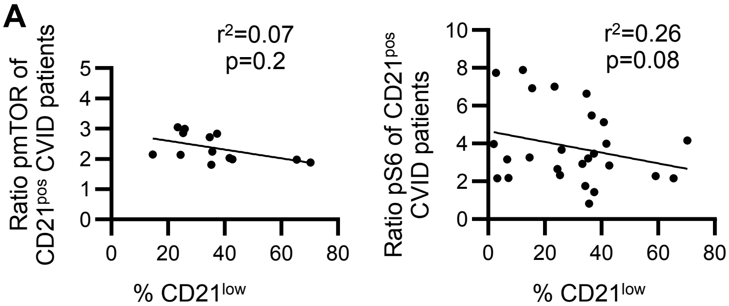

B

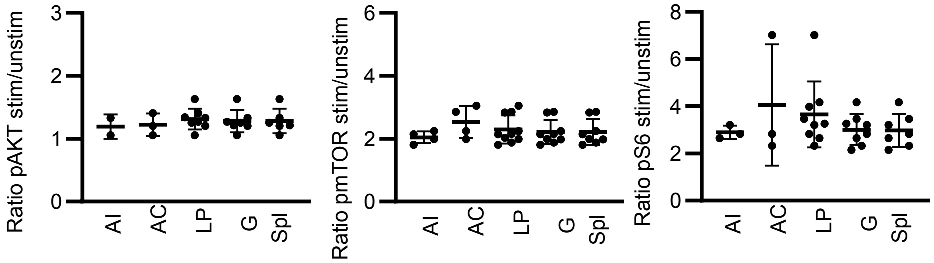

Supplementary Figure 5

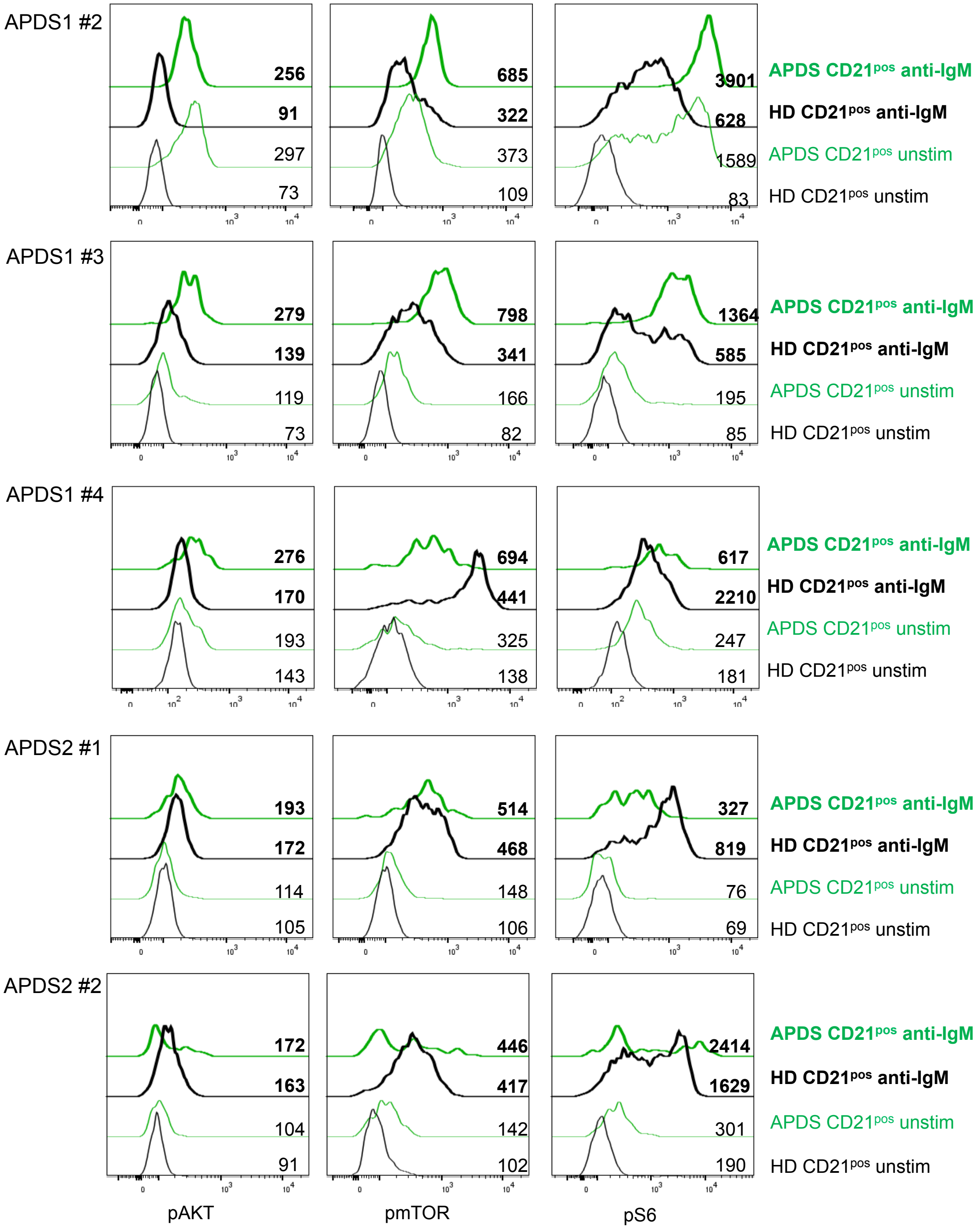

Supplement: Supplementary file 1 [file cells-11-00464-s001.zip › Supplementary.pdf]
